# Structural Changes in Monolayer Cobalt Oxides under Ambient Pressure CO and O_2_ Studied by In Situ Grazing-Incidence X-ray Absorption Fine Structure Spectroscopy

**DOI:** 10.1021/acs.jpcc.1c10284

**Published:** 2022-02-16

**Authors:** Dorotea Gajdek, Pär A. T. Olsson, Sara Blomberg, Johan Gustafson, Per-Anders Carlsson, Dörthe Haase, Edvin Lundgren, Lindsay R. Merte

**Affiliations:** † Department of Materials Science and Applied Mathematics, Malmö University, SE-211 19 Malmö, Sweden; ‡ NanoLund, Lund University, Box 118, SE-221 00 Lund, Sweden; § Division of Mechanics, Lund University, Box 118, SE-221 00 Lund, Sweden; ∥ Department of Chemical Engineering, Lund University, Box 118, SE-221 00 Lund, Sweden; ⊥ Division of Synchrotron Radiation Research, Lund University, Box 118, SE-221 00 Lund, Sweden; # Department of Chemistry and Chemical Engineering, Chalmers University of Technology, SE-412 96 Göteborg, Sweden; ∇ Competence Centre for Catalysis, Chalmers University of Technology, SE-412 96 Göteborg, Sweden; ○ MAX IV Laboratory, Lund University, Box 118, SE-221 00 Lund, Sweden

## Abstract

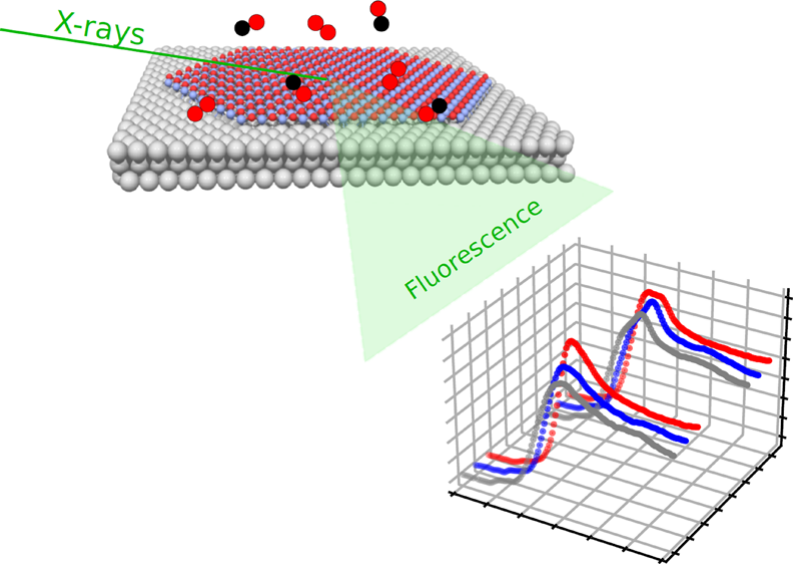

We have used grazing incidence X-ray absorption fine structure spectroscopy at the cobalt K-edge to characterize monolayer CoO films on Pt(111) under ambient pressure exposure to CO and O_2_, with the aim of identifying the Co phases present and their transformations under oxidizing and reducing conditions. X-ray absorption near edge structure (XANES) spectra show clear changes in the chemical state of Co, with the 2+ state predominant under CO exposure and the 3+ state predominant under O_2_-rich conditions. Extended X-ray absorption fine structure spectroscopy (EXAFS) analysis shows that the CoO bilayer characterized in ultrahigh vacuum is not formed under the conditions used in this study. Instead, the spectra acquired at low temperatures suggest formation of cobalt hydroxide and oxyhydroxide. At higher temperatures, the spectra indicate dewetting of the film and suggest formation of bulklike Co_3_O_4_ under oxidizing conditions. The experiments demonstrate the power of hard X-ray spectroscopy to probe the structures of well-defined oxide monolayers on metal single crystals under realistic catalytic conditions.

## Introduction

Ultrathin or two-dimensional transition metal oxides are active catalysts for several reactions and useful models for understanding the behavior of noble metal/reducible oxide catalysts, particularly where wetting of the metal occurs and where interfacial reactions are of interest.^1−4^ Supported oxides have been shown in particular to provide enhanced activity for low-temperature CO oxidation^5,6^ and the water gas shift reaction,^7^ for example.

Fundamental studies have shown that these oxides display rather complex behavior upon exposure to reactant gases by easily changing their structure. The well-studied FeO/Pt(111) system provides useful examples, including the transformation of the FeO bilayer phase to an O–Fe–O trilayer^8^ and the switching of island-edge termination via the introduction of linear defects.^9,10^ Such transformations have direct consequences for the materials’ catalytic properties and similar behavior is expected to be relevant for a variety of ultrathin oxide phases.

Cobalt oxides are active catalysts for oxidation reactions^11,12^ and form hexagonal bilayer films on Pt(111),^13−16^ Pd(100),^17^ and Au(111),^14−16^ which are similar to FeO. These CoO films exhibit similar structural changes as FeO when reduced and oxidized in high vacuum: in particular, a bilayer to CoO_2_ (or CoOOH) trilayer transformation has been identified upon oxygen exposure.^16,18^ Also, as with FeO, the edges of CoO islands were shown to be active sites for reactions such as water dissociation, and after exposure to electrochemical conditions, formation of the 2D hydroxides/oxyhydroxides Co(OH)_2_ and CoOOH was reported.^18^ Furthermore, Kersell et al.^19^ recently reported the formation of a stable carbonate at CoO island edges while exposing the film to CO and O_2_ at room temperature.

To understand the contributions of these phases to catalytic processes, it is important to be able to characterize them under reaction conditions. A common technique for such characterization is ambient-pressure X-ray photoelectron spectroscopy (XPS),^20−22^ which enables characterization of chemical states in gases and liquids in the mbar to bar pressure range. Although this technique is extremely versatile, with the capability to provide information about nearly all elements at the sample surface and in the liquid/gas phase, the information provided about specific species is usually derived solely from binding energy and thus provides only a nominal oxidation state. Furthermore, the mechanism for extraction of photoelectrons—dynamic flow conditions and differential pumping—produces a very steep pressure gradient near the surface that imposes natural limitations on the conditions and on the reaction kinetics that can be probed.

X-ray absorption fine structure spectroscopy (XAFS), performed using hard X-rays and with photon yields for detection, is a powerful method that has been applied broadly for in situ materials characterization under a wide range of conditions.^23,24^ Measurement of fine structure near the absorption edges (XANES) provides chemical fingerprints that can be used to identify specific phases, and analysis of the fine structure far above the edge—the extended X-ray absorption fine structure spectroscopy (EXAFS) region—provides structural information due to the contributions of local photoelectron scattering.^25−27^ The main limitation of the technique for surface studies is the large sampling depth of hard X-rays, which normally results in signals characteristic of the bulk material. For materials where the species of interest is found exclusively at the surface, there are no bulk contributions, and with grazing incidence geometry and fluorescence detection, sufficiently high signal-to-noise ratios can be obtained, enabling characterization of speciation and local bonding environment for submonolayer species.^28−34^

The grazing incidence X-ray absorption fine structure (GI-XAFS) technique is thus expected to be well-suited for studies of single and few-layer oxide films under in situ catalytic conditions. The main goal of this study was to demonstrate the potential of this application, using single-layer CoO_*x*_ islands grown on Pt(111) as a relevant example. These samples, grown by deposition in ultrahigh vacuum (UHV), were exposed to CO, O_2_, and a 1:1 CO/O_2_ mixture at 1 bar total pressure for several temperatures, with Co K-edge XAFS spectra acquired simultaneously. The measurements enable us to follow the chemical state of cobalt under these conditions and to gain information about the local bonding partners, bond distances, and film morphology.

## Experimental Section

### Growth of CoO Thin Films on Pt(111)

CoO film growth followed procedures reported by De Santis et al.^13^ and Fester et al.^14,15^ Sample preparation was performed at the DESY Nanolab^35^ using an ultrahigh-vacuum MBE system with low-energy electron diffraction (LEED) optics. The Pt(111) sample was cleaned by cycles of Ar^+^ sputtering, annealing in 5 × 10^–7^ mbar O_2_ at 600 °C for 10 min to remove carbon contamination, and annealing in vacuum at the same temperature to desorb oxygen. The sample temperature was measured with an optical pyrometer. The CoO thin film, with a coverage of ∼0.5 ML, was grown by electron beam evaporation of Co from a rod in 5 × 10^–7^ mbar O_2_ for 5 min. After deposition, the film was briefly annealed at 600 °C. LEED was used to confirm the formation of ordered CoO sheets, after which the sample was transported in air to the synchrotron beamline.

Testing of the growth procedure, including calibration of the evaporator and testing of the effect of air transfer on the film, was performed separately at the Department of Physics, Lund University. Scanning tunneling microscopy (STM) was used to confirm the single-layer morphology of the CoO islands and to ensure that this morphology was maintained after exposure to air for 1 h, which was approximately the time needed to transfer the sample to the catalytic cell at the XAFS beamline.

### GI-XAFS

Co K-edge (*E*_0_ = 7709 eV) GI-XAFS spectra were recorded at the P64 beamline at PETRA III, Hamburg, Germany. XANES and EXAFS spectra were collected in grazing incidence (0.5°) and out-of-plane polarization. The fluorescence signal from Co was measured using a passivated implanted planar silicon (PIPS) detector with a Fe filter. Spectra were acquired from *E*_0_ = −90 eV to *E*_0_ = +610 eV in continuous-scanning mode. Several scans were acquired for each condition and averaged after checking for artifacts. The total acquisition time for one condition was ∼1 h. A Co metallic foil spectrum was collected simultaneously to perform energy calibration. Transmission-mode reference spectra for Co_3_O_4_ and CoO were provided by the beamline staff, and a spectrum for Co(OH)_2_ was taken from the Lytle database.^36^

XAFS data processing and analysis were performed using the Larch^37^ package, with EXAFS fits performed using the implementation of IFEFFIT^38^ included in that package. Scattering paths were simulated using FEFF9.6.^39^ Co–O and Co–Co paths used for fitting were simulated using rock-salt CoO, and Co–Pt paths were simulated using the structure of the CoO bilayer as found by density functional theory (DFT) relaxation. Simulated EXAFS spectra for different structures were obtained by averaging single-site spectra generated by FEFF, using either DFT-relaxed structures or models exhibiting simplified geometries that were generated manually using the atomic simulation environment (ASE).^40^ For the CoO_2_ trilayer phase, simulated by DFT within the same (9 × 9) unit cell as the bilayer, a subset of 12 Co atoms in 6-fold oxygen coordination was used.

**Figure 1:**
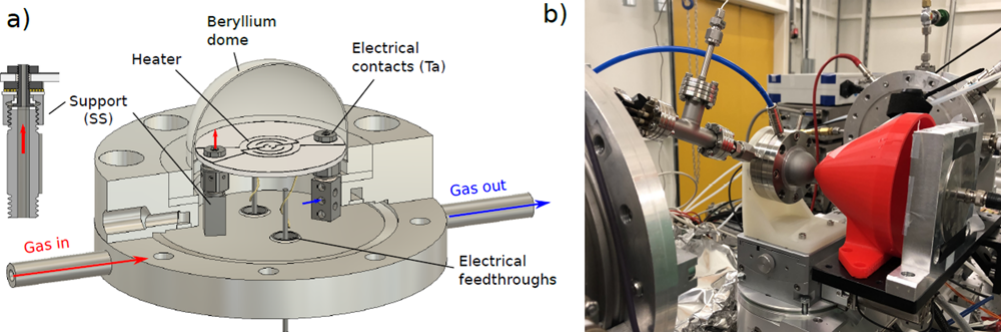
(a) Schematic of the gas cell used for XAFS measurements. (b) Photo of the cell in the configuration used for measurements.

The in situ XAFS measurements made use of a custom-built catalytic cell based on a hemispherical beryllium dome. A schematic of the cell, together with a photo of the cell in the configuration setup used for measurements, is shown in Figure 1. The cell incorporates a pyrolytic graphite/pyrolytic boron nitride heater upon which the sample is directly placed. The sample temperature was measured with a thermocouple placed at the back side of the crystal through a hole in the heater. The gas inlet is via a hole in one of the heater support legs, while the main outlet is via larger holes at the base of the other support leg (see Figure 1a). Therefore, the gas is introduced directly into the volume above the heater and extracted from beneath to reduce the potential for contamination via contact of the gas with various materials below the heater. Auger electron spectroscopy was performed after the experiment and revealed no detectable contamination aside from carbon.

**Figure 2:**
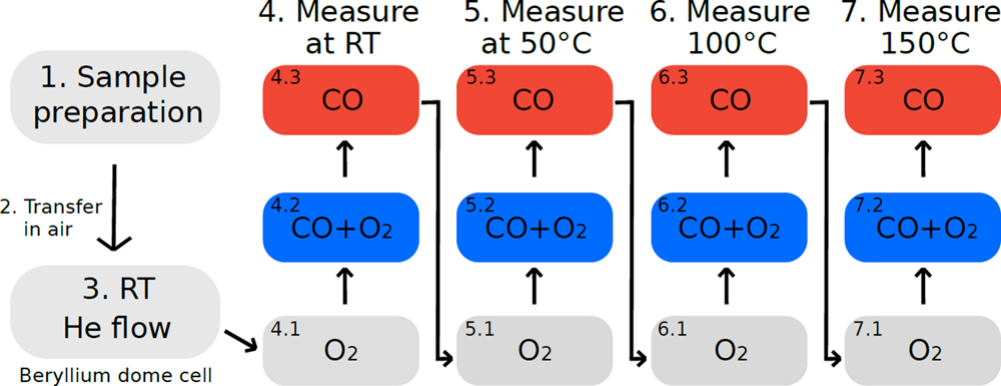
Sequence of measurements in the performed experiment.

Gases used were CO and O_2_, each diluted to 5% in He mixed either 1:1 with each other or with He to give total concentrations of 2.5%. All gases had a purity grade of at least N4.6. The sequence of the conditions tested is shown in Figure 2. A carbonyl trap was used on the CO line to prevent metal contamination. The total pressure was set to 1 bar, giving partial pressures of ca. 25 mbar for each gas. Total gas flow during the experiments was set to 50 mL̇/min, controlled with individual mass flow controllers (Bronkhorst) for each gas. He gas was used to flush the cell before and after measurements. A quadrupole mass spectrometer (Pfeiffer PrismaPlus) was used to measure the composition of the exhaust gas from the cell, which was sampled via an adjustable leak valve.

### Density Functional Theory

To produce realistic models of the Co oxide phases for EXAFS simulations, we resorted to DFT + *U* modeling. To this end, we used the Vienna ab initio simulation package (VASP).^41−44^ The interaction between valence electrons and the core was described using standard pseudopotentials from the VASP library, generated with the projector augmented-wave (PAW) method.^45,46^ The electronic valence configurations were 3d^8^4s^1^ (Co), 2s^2^2p^4^ (O), and 5d^9^6s^1^ (Pt), and the exchange correlation functional was described within the generalized gradient approximation within the Perdew–Wang (PW91) formalism.^47^ To account for the magnetic properties and the strong electronic correlation between the Co 3d-states, we utilized a collinear spin-modeling approach in conjunction with the rotationally invariant Hubbard correction approach by Dudarev et al.^48^ The effective Hubbard parameter, *U*_eff_, which is the difference between the on-site Coulombic parameter (*U*) and the effective on-site exchange parameter (*J*), was chosen as *U* – *J* = 4.0 eV, such that it reproduced the experimental bandgap of the bulk antiferromagnetic spin type II rocksalt CoO phase.^49^ The same structure was used to converge the plane wave kinetic energy cutoff, for which it was found that a cutoff of 650 eV rendered well-converged results.

To model the layer and substrate, we used an 8 × 8 CoO_*x*_ layer on a three-layered 9 × 9 (111) Pt substrate. We utilized a vacuum interface corresponding to 12 Å between the top and bottom layers along with a dipole correction to reduce the artificial interaction across the vacuum.^50^ Owing to the large size and 2D nature of the system, the reciprocal space was discretized using the Γ-point as the only *k*-point. To relax the structure, we allowed the CoO_*x*_ layer and the topmost Pt layer to undergo full coordinate relaxation using a two-step procedure where we first used a quasi-Newton algorithm, followed by damped molecular dynamics relaxation. This procedure resulted in a stable relaxation scheme.

## Results

### Grazing Incidence XAFS

**Figure 3:**
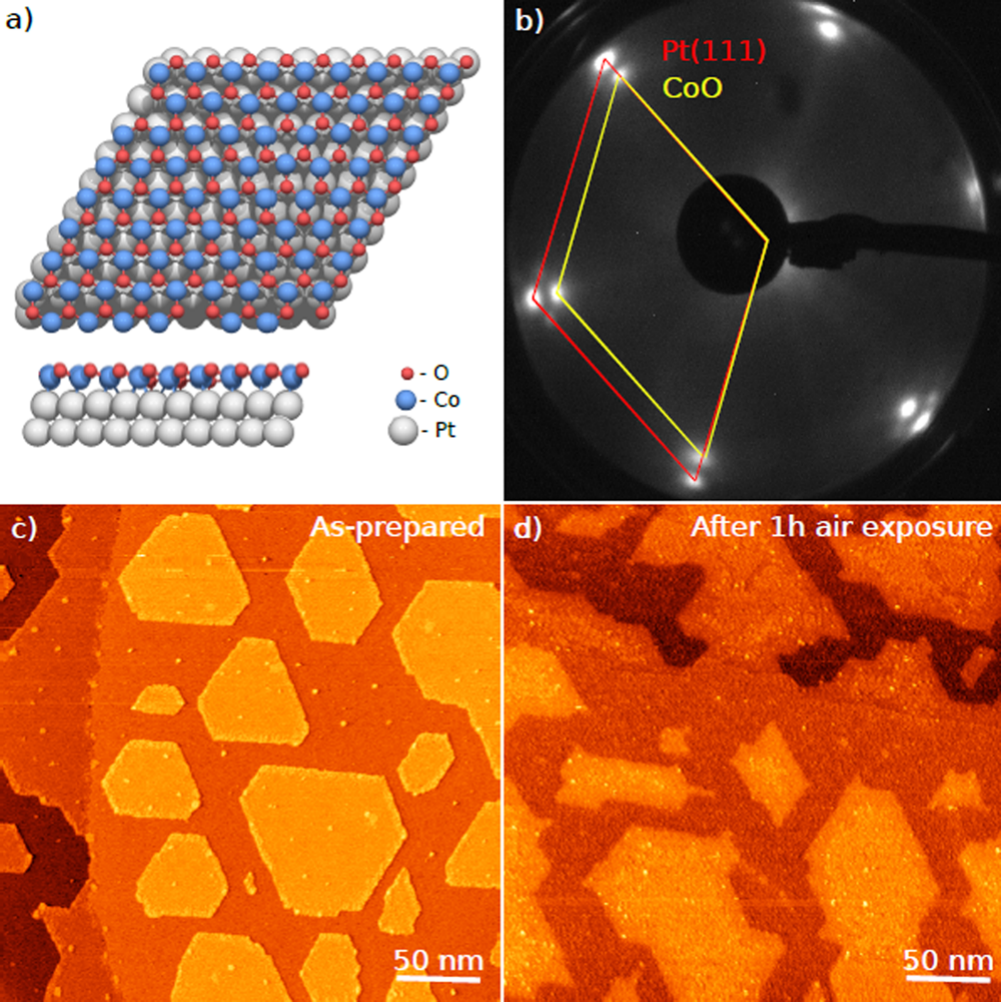
CoO thin films on Pt(111). (a) Ball model of CoO bilayer on Pt(111) substrate. (b) LEED image (50 eV) recorded at DESY NanoLab before XAFS measurements. (c) STM image recorded under constant-current mode of an as-prepared CoO thin film observed as islands on Pt(111) substrate. (d) STM image of the surface after 1 h air exposure.

The CoO film grown on Pt(111) exhibits a hexagonal monolayer structure with a characteristic moiré pattern as shown in Figure 3a. The film grown immediately before XAFS experiments was characterized by LEED (Figure 3b). STM measurements (Figure 3c and d) performed separately show that CoO forms monolayer-thick islands under these conditions, as expected, and that exposure to air does not alter the monolayer morphology of the islands.

**Figure 4:**
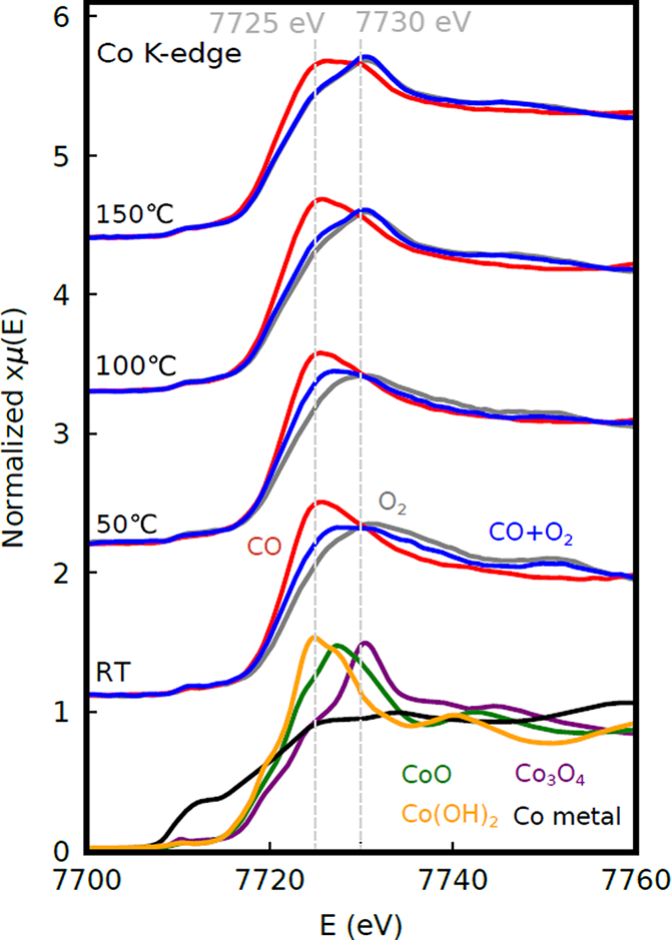
XANES spectra recorded from CoO_*x*_/Pt(111) during CO (red), O_2_ (gray), and CO +O_2_ (blue) flows at four different temperatures together with reference spectra of CoO, Co_3_O_4_, Co(OH)_2_, and Co metal. Dashed lines mark the expected positions for peaks due to Co^2+^ and Co^3+^, respectively.^51−53^

Co K-edge XANES spectra from the CoO_*x*_/Pt(111) sample are shown in Figure 4. The spectra show clear changes depending on the gas composition at all applied temperatures. At room temperature (RT), we observe a shift in the white line maximum position between about 7725 and 7730 eV for pure CO and pure O_2_, respectively, with an intermediate energy observed in the CO + O_2_ mixture. These changes indicate changes in the oxidation state of Co, presumably between Co^2+^ and Co^3+^.^51−53^ The spectra obtained at 50 °C are similar to those at RT. Significant differences are observed at higher temperatures, however; the white lines become sharper, and the spectra acquired in O_2_ exhibit a sharp peak at 7730 eV with a distinct low-energy shoulder, characteristic of Co_3_O_4_,^51−53^ which contains a mixture of Co^2+^ and Co^3+^. At these temperatures, the spectra acquired in the CO + O_2_ mixture are very similar to those in pure O_2_. Little difference is observed between 100 and 150 °C, with the most marked being a flattening of the white line in CO. The formation of metallic Co was not observed under any conditions.

**Figure 5:**
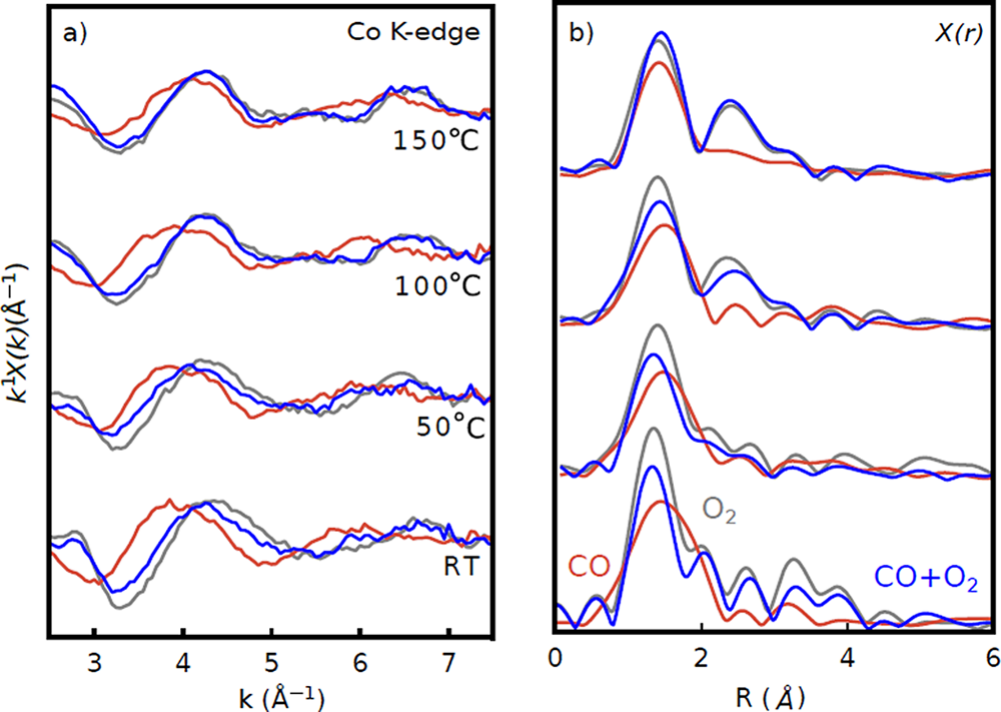
(a) *k*^1^ weighted EXAFS spectra of CoO_*x*_/Pt(111) during CO (red), O_2_ (gray), and CO + O_2_ (blue) exposure at four different temperatures (RT and 50, 100, and 150 °C). (b) Fourier transforms of the spectra in (a).

Although limited by the relatively low signal caused by the low concentration of Co in these samples, EXAFS oscillations up to *k* ≈ 7.5 Å^–1^ could be extracted. These data are plotted in Figure 5 and show distinct and reproducible changes for different gas compositions and temperatures. The spectra are dominated by components at ∼1.4 Å (expected for Co–O scattering), with a shift in position between oxidizing and reducing conditions at low temperatures. An additional component at ∼2.5 Å appears at higher temperatures.

**Figure 6:**
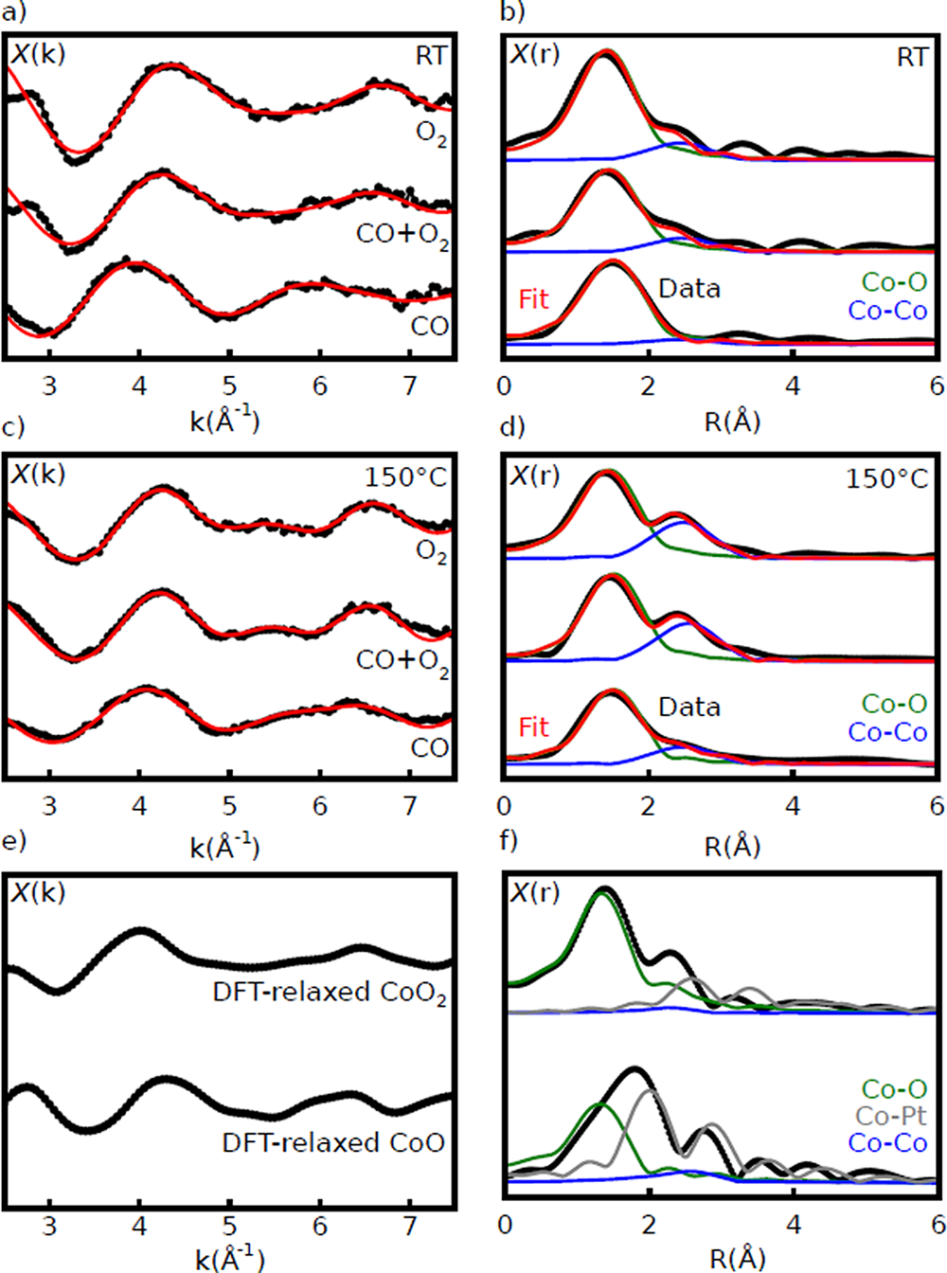
Raw EXAFS spectra and fits with specific scattering-path contributions for all three gas flows at RT (a, b) and 150 °C (c, d) and for DFT-relaxed CoO and DFT-relaxed CoO_2_ (e, f).

The limited range prevents full quantitative structural analysis, particularly due to the correlation between Debye–Waller parameters and coordination numbers, but identification of nearest neighbors and estimation of bond lengths is possible. Figure 6 shows fits for all three gas mixtures at RT and 150 °C. Reasonable fits for all spectra could be obtained using a combination of Co–O and Co–Co paths, with the latter being responsible for the 2.5 Å component at higher temperatures. Inclusion of Co–Pt scattering paths did not lead to reasonable fits for any of the spectra. Co–O bond lengths were found to vary between 1.93 Å for oxidizing conditions to 2.07 Å under CO at room temperature. Co–Co distances observed under oxidizing conditions at 100 and 150 °C, where these contributions were the strongest, were ∼2.9 Å. Full details of the fit results are presented in the Supporting Information.

### EXAFS Simulations

**Figure 7:**
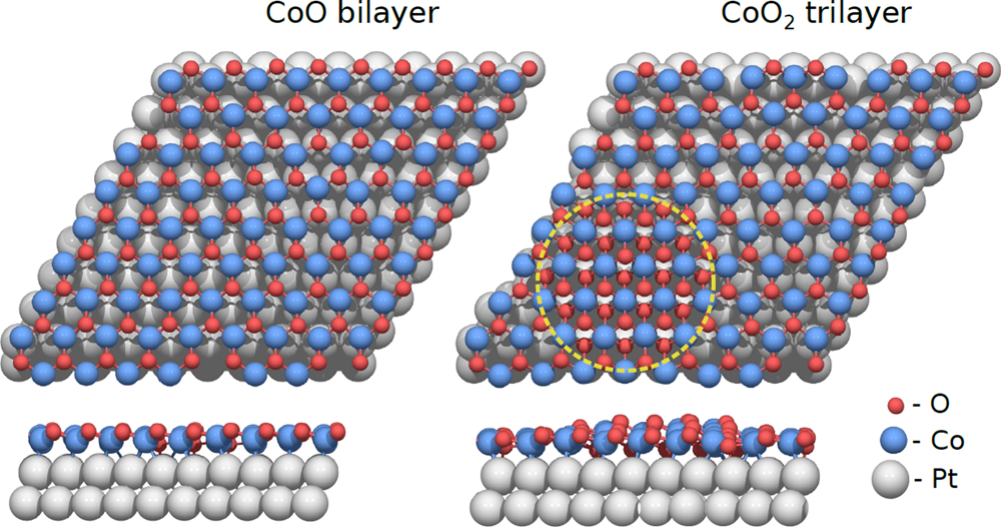
Top and side views of a CoO bilayer structure (left) and a CoO_2_ trilayer structure (right, indicated by circle) after optimization with DFT.

Interpretation of the obtained EXAFS data requires comparison with expected features from model structures. As discussed earlier, on the basis of previous UHV experiments,^16,18^ the anticipated model for evolution of the CoO_*x*_ structure under reducing and oxidizing conditions involves a transition between the CoO bilayer and CoO_2_ trilayer phases, as illustrated in Figure 7, which shows DFT-relaxed structures for the moiré-type bilayer and a model for the CoO_2_ trilayer where an extra O layer has been added at the HCP domain in the same moiré cell.

**Figure 8:**
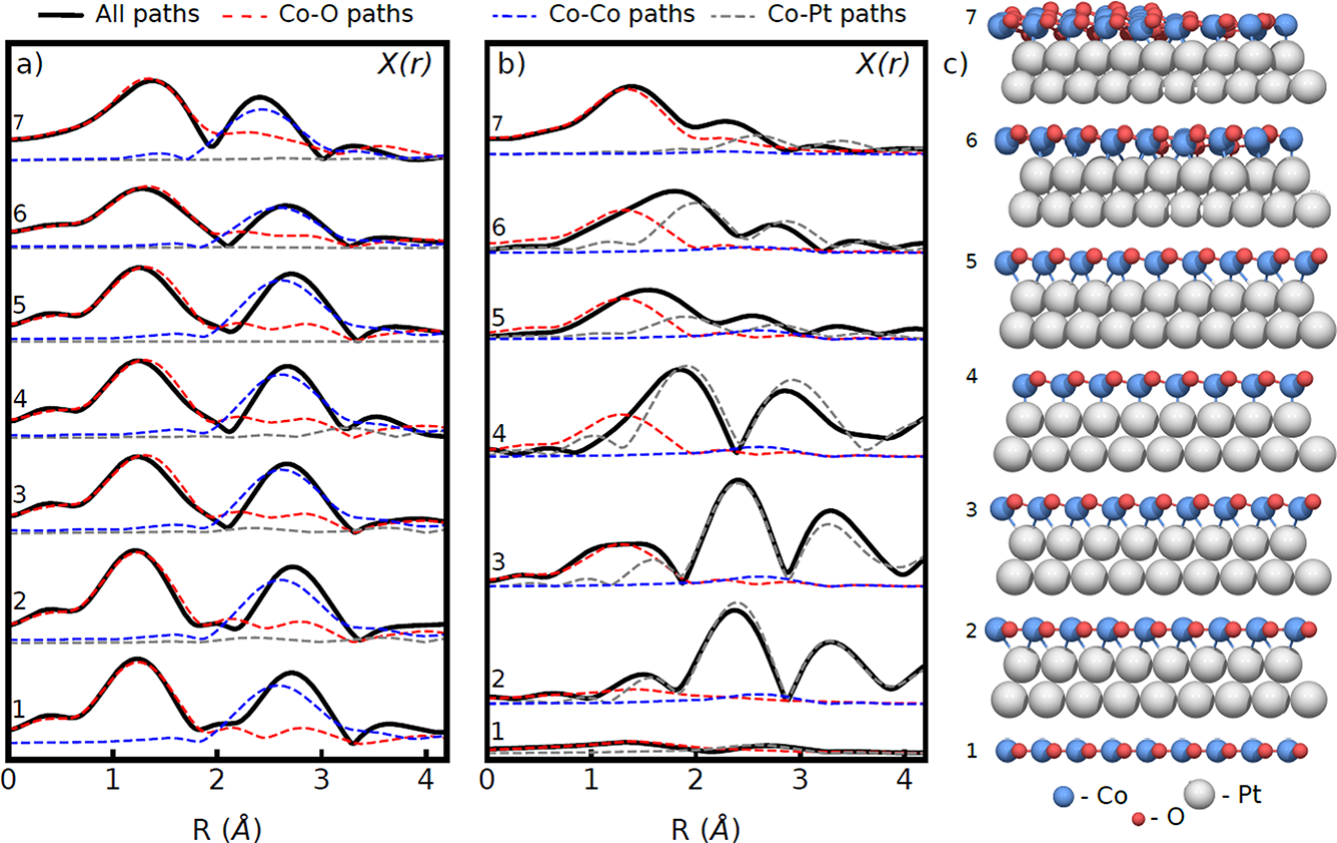
Simulated EXAFS spectra for various model CoO structures. (a) In-plane polarization. (b) Out-of-plane polarization. (c) Corresponding models. Structures: (1) flat, unsupported CoO; (2) flat, supported CoO/Pt(111); (3) buckled, supported CoO/Pt(111) with CoO lattice matched to that of Pt(111) face-centered cubic (fcc) stacking; (4) buckled, supported CoO/Pt(111) with CoO lattice matched to that of Pt(111) on-top stacking; (5) buckled, supported CoO lattice with moiré pattern but no local relaxation; (6) DFT-relaxed CoO/Pt(111) bilayer; and (7) DFT-relaxed CoO_2_/Pt(111) trilayer.

To illustrate the relationship between structural features and their corresponding features in the EXAFS spectra, we simulated spectra for a series of structures approximating that of the CoO bilayer, along with the optimized DFT structures. These simulated spectra are plotted in Figure 8. The spectra simulated for in-plane polarization, not measured experimentally, all show consistent features, i.e., strong Co–O and Co–Co scattering nearly independent of the structural variations. In contrast, the out-of-plane spectra, which were measured experimentally, show clear changes linked to different structural features.

The simplest model for the CoO sheet is a flat, unsupported monolayer (no. 1 in Figure 8) with h-BN structure. EXAFS for this structure shows very weak scattering for out-of-plane polarization, consistent with the absence of scatterers in this direction. Inclusion of the Pt(111) support, with an expanded lattice matching that of the oxide layer (no. 2), leads to the appearance of Co–Pt scattering features due to interfacial bonding. The introduction of interlayer buckling (no. 3) by taking the mean value of the Co–O layer separation from the DFT-relaxed structure, leads to the appearance of a Co–O scattering component.

Models 3 and 4 show clearly the strong impact of the Co–Pt scattering components for these pseudomorphic structures; shifting the atoms from above the hollow sites (no. 3) to sites directly atop the Pt atoms (no. 4) with the same mean height above the surface leads to a strong shift in the Co–Pt scattering length. In the absence of relaxation, the strong variation in the Co–Pt distance in different domains of the moiré structure leads to strongly dampened EXAFS features, as seen in model no. 5. Local relaxation in the DFT structure (no. 6) leads to more uniform Co–Pt distances across the moiré cell and results in strengthening of the Co–Pt components.

The expected EXAFS spectrum for the CoO bilayer (no. 6) therefore exhibits a combination of Co–O and Co–Pt scattering features. Oxidation of the phase to form the CoO_2_ trilayer (no. 7) yields a strengthening of the Co–O component and a weakening of the Co–Pt component, resulting in a spectrum dominated by Co–O scattering. Note that Co–Co scattering is not expected for any of the structures due to the Co atoms being nearly coplanar.

## Discussion

The changes observed in XANES and EXAFS spectra with increasing temperature—changing white line profiles and the appearance of Co–Co scattering—are consistent with a dewetting transition from an initial 2D layer to 3D islands. The spectra acquired at 100 and 150 °C are consistent with bulk-like Co_3_O_4_. This spinel compound exhibits first-shell (Co–O) distances of 1.92 Å and second-shell (Co–Co) distances of 2.86 Å, which are close to those measured here (1.96 ± 0.02 and 2.89 ± 0.02 Å, respectively, in O_2_ at 150 °C). In CO gas at these temperatures, where the oxide shows XANES features indicative of Co^2+^, the Co–Co component is significantly weaker, apparently indicating a flattening of the oxide under these conditions. We note that dewetting under CO oxidation conditions was also observed previously for FeO/Pt(111) by Sun et al.^5^

The XANES spectra acquired at low temperatures clearly show oxidation and reduction under O_2_ and CO, in line with expectations for the CoO ↔ CoO_2_ transition. The EXAFS spectra, however, indicate that this process does not take place under the conditions of our experiment. In particular, the absence of Co–Pt scattering contributions, the dominance of the Co–O component, and the length of the Co–O bonds (∼2.05 vs 1.9 Å in the bilayer structure according to DFT) enable us to rule out the presence of the CoO bilayer in CO.

The behavior of the low-temperature spectra is in fact somewhat surprising: despite large changes in the XANES region, we see rather little difference in EXAFS, excepting the elongation of Co–O bonds from ∼1.93 to ∼2.05 Å when going from O_2_ to CO. The behavior could be explained by the formation of cobalt hydroxides and oxyhydroxides. Cobalt forms the double hydroxide compound Co(OH)_2_, which incorporates Co^2+^ in octahedral coordination and thus should exhibit EXAFS features similar to those of the CoO_2_ trilayer. Under oxidizing conditions this can be converted to the oxyhydroxide CoOOH with similar coordination. Simulated EXAFS spectra for these compounds are shown in the Supporting Information and are essentially the same as that calculated for CoO_2_. The Co–O bond lengths in Co(OH)_2_ and CoOOH are 2.09 and 1.90 Å,^54^ respectively, which seems consistent with our measurements. The spectral features that we observed are also consistent with those of cobalt carbonates,^19^ although such a phase was found to be stable only at the edges of CoO islands and probably did not make a substantial contribution in our case (STM indicates that our growth recipe leads to islands ∼50–100 nm in diameter). Simulated EXAFS for such a phase is included in the Supporting Information as well.

The presence of cobalt hydroxides is to be expected under humid or aqueous conditions, and the transformation between Co(OH)_2_ and CoOOH has been reported previously for CoO on Au(111) exposed to aqueous electrochemical conditions.^55^ Although we used dry gases in our experiments, it has been shown that trace quantities of water or hydrogen can be sufficient to form hydroxides. Fester et al.^56^ reported the formation of CoOOH after growth of 2D cobalt oxides on Au(111) under ultrahigh-vacuum conditions, with hydrogen apparently adsorbing from the chamber background gas. Similar behavior was observed for oxidized iron oxide films on Pt(111) under UHV and near-ambient pressure conditions.^22,57^ We thus find it plausible that trace water in the cell or gas lines would be sufficient to lead to hydroxide formation. Because the transformation was found to be reversible, we do not believe that the transfer through air was decisive. The change in behavior of the material at 100 and 150 °C compared to that at lower temperatures may be attributable to the thermal decomposition of hydroxides and the formation of oxides instead. Nevertheless, the bilayer phase was not observed under these conditions either, as the film seemed to be unstable against dewetting.

Although our setup was insufficiently sensitive to detect low-temperature CO oxidation, which is presumed to occur at the edges of CoO_*x*_ islands, under these conditions the spectra nevertheless indicate operation of a redox process. At the lower temperatures, the observation of spectra showing an intermediate average oxidation state indicates that the oxidation and reduction processes occur at similar rates, while at higher temperatures the oxidation process dominates. Our assignment of the low-temperature phases to Co hydroxide and oxyhydroxides implies half-reactions involving water,
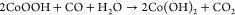

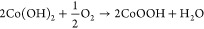
suggesting that reactions involving hydroxyl groups as well as proton-transfer steps must be accounted for in gas-phase CO oxidation by Pt-supported CoO_*x*_ catalysts. This is consistent with previous studies of nanoparticle-based catalysts suggesting high reactivity of hydroxyl groups in low-temperature CO oxidation as well as electrochemical reactions like OER.^58,59^

The XANES spectra acquired in these experiments were found to be of good quality and showed strong sensitivity to changes in the chemical state of ultrathin oxides under ambient-pressure reaction conditions. This can provide an advantage in comparison to, e.g., ambient-pressure XPS, where often only the oxidation state can be determined. The inclusion of EXAFS data as well adds further to the value of GI-XAFS, and these experiments have demonstrated the importance of structural information to the interpretation of the results. Nevertheless, the limited range of useful data here was somewhat dissatisfying, considering the usual expectations for analysis of bulk samples. We see room for improvement, however; the noise that limited our analysis range was not random but rather appeared to be caused by diffraction from the single-crystal platinum substrate. Use of an energy-discriminating detector should reduce these contributions. Test measurements using a silicon-drift detector have shown clean spectra with useful signals for similar samples up to at least *k* = 10 Å^–1^. We thus anticipate these types of experiments to be very valuable for studies of ultrathin films under catalytic or electrocatalytic conditions.

## Conclusions

Using grazing incidence XAFS at the Co K-edge, we were able to follow changes in chemical state and local structure for monolayer cobalt oxides on Pt(111) under ambient-pressure CO oxidation conditions at temperatures up to 150 °C. The spectra indicate that Co is present in either the Co^2+^ state or the Co^3+^ state depending on the gas composition and that reduction to CO metal does not take place under these conditions.

The spectra allow us to rule out the presence of bilayer CoO under CO and instead point toward 2D hydroxides or oxyhydroxides as the dominant phases at low temperatures, with hydrogen likely emerging from trace water in the system. At higher temperatures, we see evidence that the oxides dewet the surface, and under oxidizing conditions the spectra appear consistent with Co_3_O_4_.

The results demonstrate the value of GI-XAFS for in situ studies of well-defined, single-crystal supported 2D oxides under ambient-pressure conditions, particularly when performed at high-brilliance synchrotron beamlines. We anticipate further improvements in data quality, which will enable more detailed characterization of such phases in the future.

## Supporting Information Available

Full description of EXAFS fit results for measured data as well as simulated EXAFS spectra for hydroxide, oxyhydroxide, and carbonate structures (PDF)
