# Supplementary material for: Structural Changes in Monolayer Cobalt Oxides under Ambient Pressure CO and O2 Studied by In Situ Grazing-Incidence X-ray Absorption Fine Structure Spectroscopy
Source: J Phys Chem C Nanomater Interfaces. 2022 Feb 16;126(7):3411–8. doi: 10.1021/acs.jpcc.1c10284 (PMC8883796; doi:10.1021/acs.jpcc.1c10284)
Supplement: Supplementary file 1 — jp1c10284_si_001.pdf [file jp1c10284_si_001.pdf]

Supporting Information

Structural Changes in Monolayer Cobalt Oxides under Ambient Pressure CO and O_2 Studied by In Situ Grazing-Incidence X-ray Absorption Fine Structure Spectroscopy

Dorotea Gajdek (1,2), Pär A. T. Olsson (1,3), Sara Blomberg (4,2), Johan Gustafson (5), Per-Anders Carlsson (6,7), Dörthe Haase (8), Edvin Lundgren (5,2), Lindsay R. Merte*(1,2)

(1) Department of Materials Science and Applied Mathematics, Malmö University, SE-211 19 Malmö, Sweden
(2) NanoLund, Lund University, Box 118, SE-211 00, Lund, Sweden
(3) Division of Mechanics, Lund University, Box 118, SE-221 00 Lund, Sweden
(4) Department of Chemical Engineering, Lund University, Box 118, SE-221 00 Lund, Sweden
(5) Division of Synchrotron Radiation Research, Lund University, Box 118, SE-221 00 Lund, Sweden
(6) Department of Chemistry and Chemical Engineering, Chalmers University of Technology, SE-412 96, Göteborg, Sweden
(7) Competence Centre for Catalysis, Chalmers University of Technology, SE-412 96 Göteborg, Sweden
(8) MAX IV Laboratory, Lund University, Box 118, SE-221 00 Lund, Sweden

*lindsay.merte@mau.se

EXAFS fit results for measured data

Table S1: EXAFS fitting parameters for measured data for all reaction conditions. R-range used for fitting was 1.0-4.5 Å and k-range used was 2.5-7.5 Å^{-1}.

T / °C	Gas flow	Path	N	R(Å)	σ^2(Å^2)	ΔE(eV)
RT	O_2	Co-O	5.5 (0.5)	1.93 (0.03)	*0.008*	-5.1 (2.6)
		Co-Co	1.96 (0.9)	2.85 (0.06)	*0.008*	
	CO + O_2	Co-O	4.4 (0.4)	1.97 (0.03)	*0.008*	-5.2 (2.9)
		Co-Co	1.7 (0.8)	2.87 (0.06)	*0.008*	
	CO	Co-O	5.2 (0.3)	2.06 (0.02)	*0.008*	-7.0 (1.7)
		Co-Co	0.51 (0.5)	2.83 (0.08)	*0.008*	
50	O_2	Co-O	4.9 (0.3)	1.97 (0.02)	*0.008*	-4.3 (1.8)
		Co-Co	2.1 (0.7)	2.96 (0.04)	*0.008*	
	CO + O_2	Co-O	3.8 (0.3)	1.98 (0.03)	*0.008*	-8.0 (2.6)
		Co-Co	1.1 (0.6)	2.88 (0.06)	*0008*	
	CO	Co-O	4.2 (0.3)	2.07 (0.03)	*0.008*	-6.6 (2.3)
		Co-Co	1.0 (0.6)	2.96 (0.07)	*0.008*	
100	O_2	Co-O	4.5 (0.2)	1.95 (0.01)	*0.008*	-5.2 (1.3)
		Co-Co	3.5 (0.4)	2.88 (0.02)	*0.008*	
	CO + O_2	Co-O	4.0 (1.2)	1.97 (0.01)	*0.008*	-4.8 (1.3)
		Co-Co	3.2 (0.4)	2.89 (0.02)	*0.008*	
	CO	Co-O	3.9 (0.3)	2.05 (0.03)	*0.008*	-8.0 (2.3)
		Co-Co	0.5 (0.6)	3.00 (0.11)	*0.008*	
150	O_2	Co-O	4.1 (0.2)	1.96 (0.02)	*0.008*	-4.8 (1.4)
		Co-Co	3.9 (0.4)	2.89 (0.02)	*0.008*	
	CO + O_2	Co-O	3.5 (0.5)	2.00 (0.02)	*0.008*	-1.95 (1.6)
		Co-Co	3.4 (0.8)	2.92 (0.02)	*0.008*	
	CO	Co-O	2.7 (0.5)	2.03 (0.02)	*0.008*	-5.3 (1.8)
		Co-Co	1.1 (0.3)	2.89 (0.03)	*0.008*	

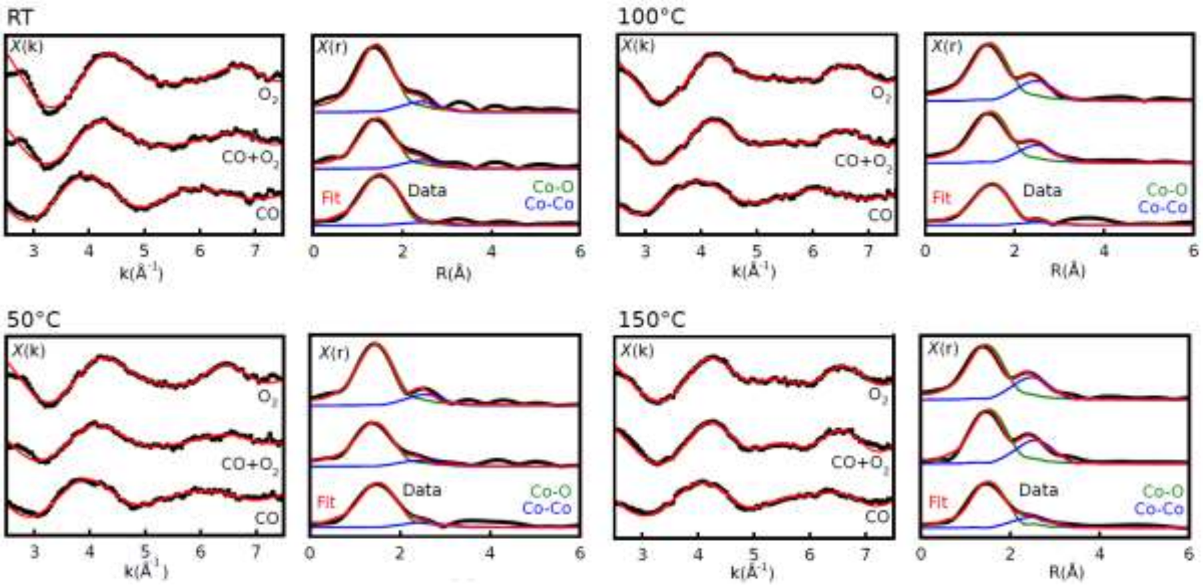

Figure S1: k^1 weighted EXAFS spectra of CoO_x/Pt(111) during exposure to O_2, CO+O_2 and CO flows at room temperature (RT), 50°C, 100°C and 150°C with fits.

Simulated hydroxide, oxy-hydroxide and carbonate structures

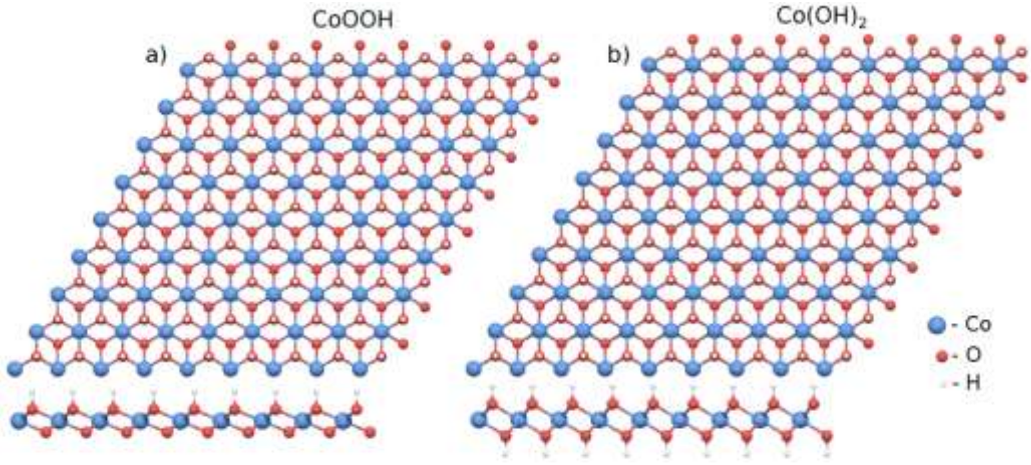

Figure S2: a) CoOOH monolayer, top and side view. Co-O distance of 1.897 Å and O-H distance of 1.248 Å as found by Delaplane at al. (1) **b)** $Co(OH)_2$ monolayer, top and side view. Co-O distance of 2.097 Å and O-H distance of 1.248 Å as found by Lotmar at al. (2).

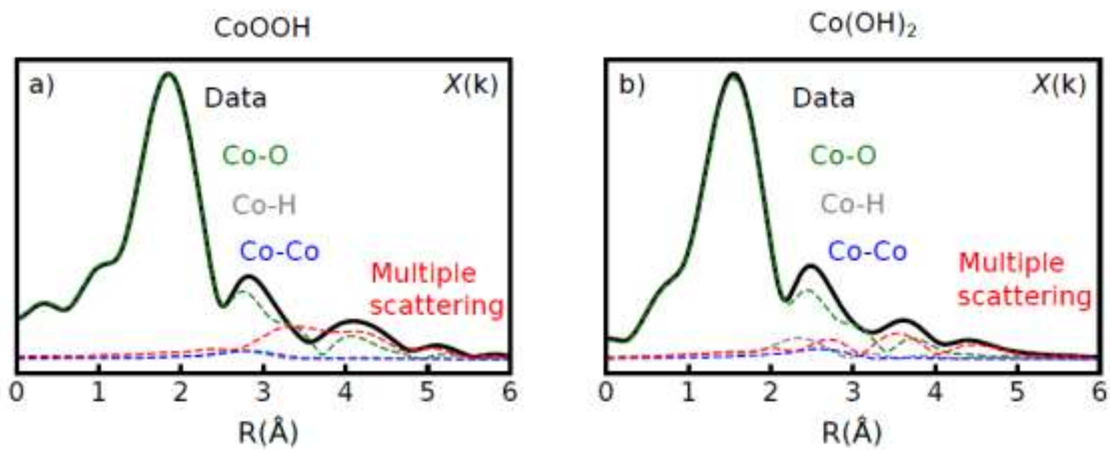

Figure S3: Fourier transformed k[1] weighted EXAFS spectra for a) CoOOH and b) $Co(OH)_2$ showing individual Co-O, Co-Co, Co-H and multiple-scattering path contributions.

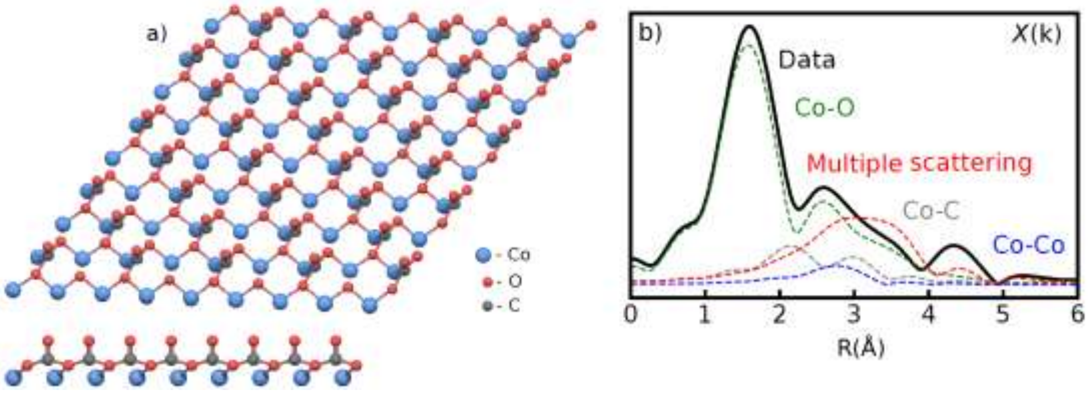

Figure S4: Modeled monolayer cobalt carbonate **a)** $CoCO_3$ ball model, angled top and side view. Assumed the adsorption mechanism of CO_2 onto CoO_x adapted from Kersell et al. (3) with a Co-O distance of 2.109 Å as found by Pertlik. (4) **b)** Fourier transformed k[1] weighted EXAFS spectrum showing individual Co-O, Co-Co, Co-C and multiple-scattering path contributions.

References

1. Delaplane, R. G., Ibers, J. A., Ferraro, J. R. and Rush, J. J.: Diffraction and spectroscopic studies of the cobaltic acid system HCoO 2-DCoO2. *J. Chem. Phys.* **1969**, 50**,** 1920-1927.
2. Lotmar, W. and Feitknecht, W.: Über Änderungen der Ionenabstände in Hydroxyd-Schichtengittern. *Z. Kristallogr. Cryst. Mater* **1936**, 93**,** 368.
3. Kersell H, Hooshmand Z., Yan G., Le D., Nguyen H., Eren B., Hao Wu C., Walauyo I., Hunt A., Nemšák S., Somorjai G., Rahman T. S., Sautet P. and Salmeron M.: CO Oxidation Mechanisms on CoOx-Pt Thin Films. *J. Am. Chem. Soc.* **2020**, 142, 8312–8322.4. F., Pertlik.: Structures of hydrothermally synthesized cobalt(II) carbonate and nickel(II) carbonate. *Acta Cryst. C* **1986**, 42**,** 4-5.
